# Supplementary figures and images for: Ecophysiology of the Cosmopolitan OM252 Bacterioplankton (Gammaproteobacteria)
Source: mSystems. 2021 Jun 29;6(3):e00276-21. doi: 10.1128/mSystems.00276-21 (PMC8269220; doi:10.1128/mSystems.00276-21)

Figure S1

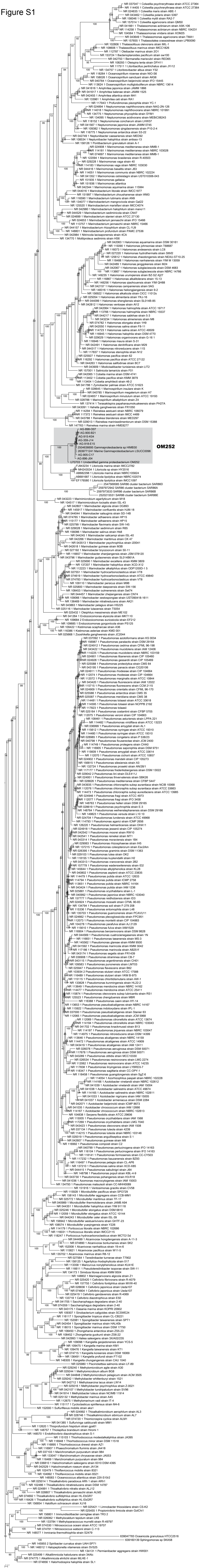

Supplement: FIG S1 [file msystems.00276-21-sf001.pdf]

Figure S2

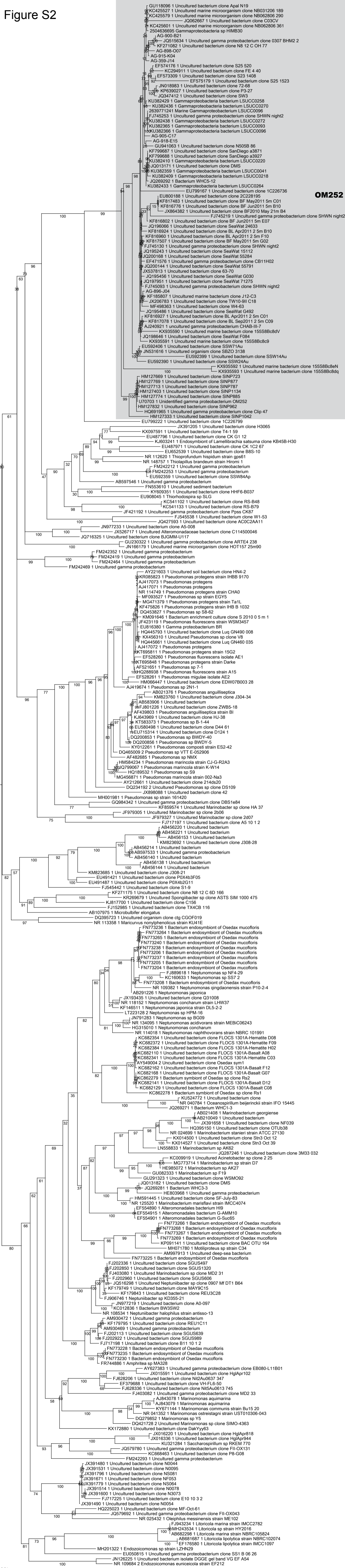

0.01

Supplement: FIG S2 [file msystems.00276-21-sf002.pdf]

Figure S3

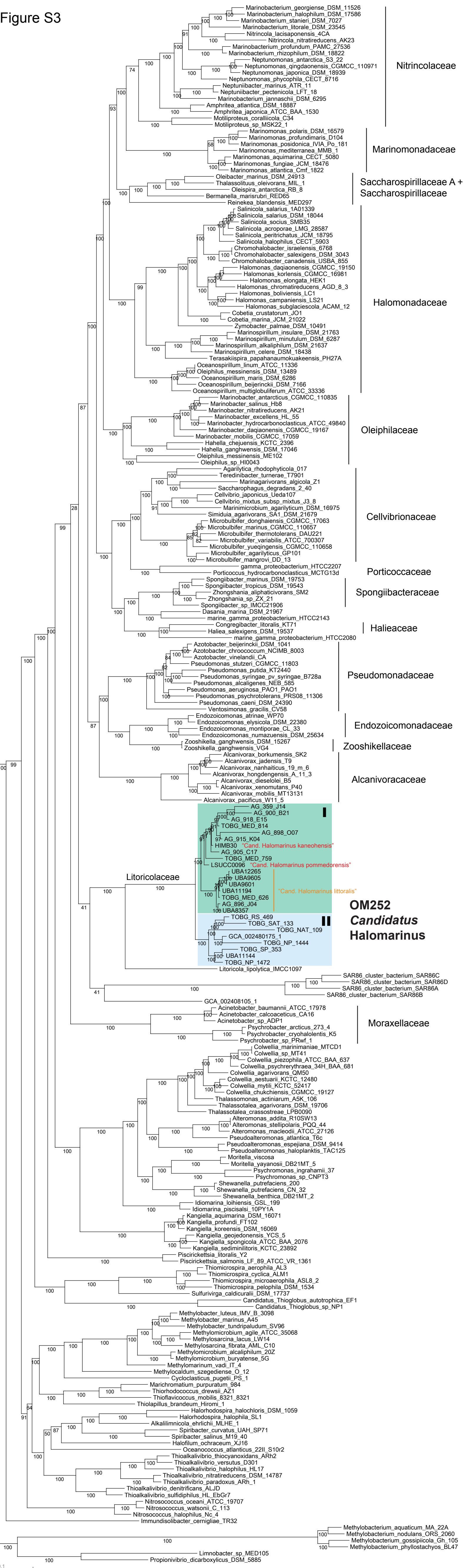

Supplement: FIG S3 [file msystems.00276-21-sf003.pdf]

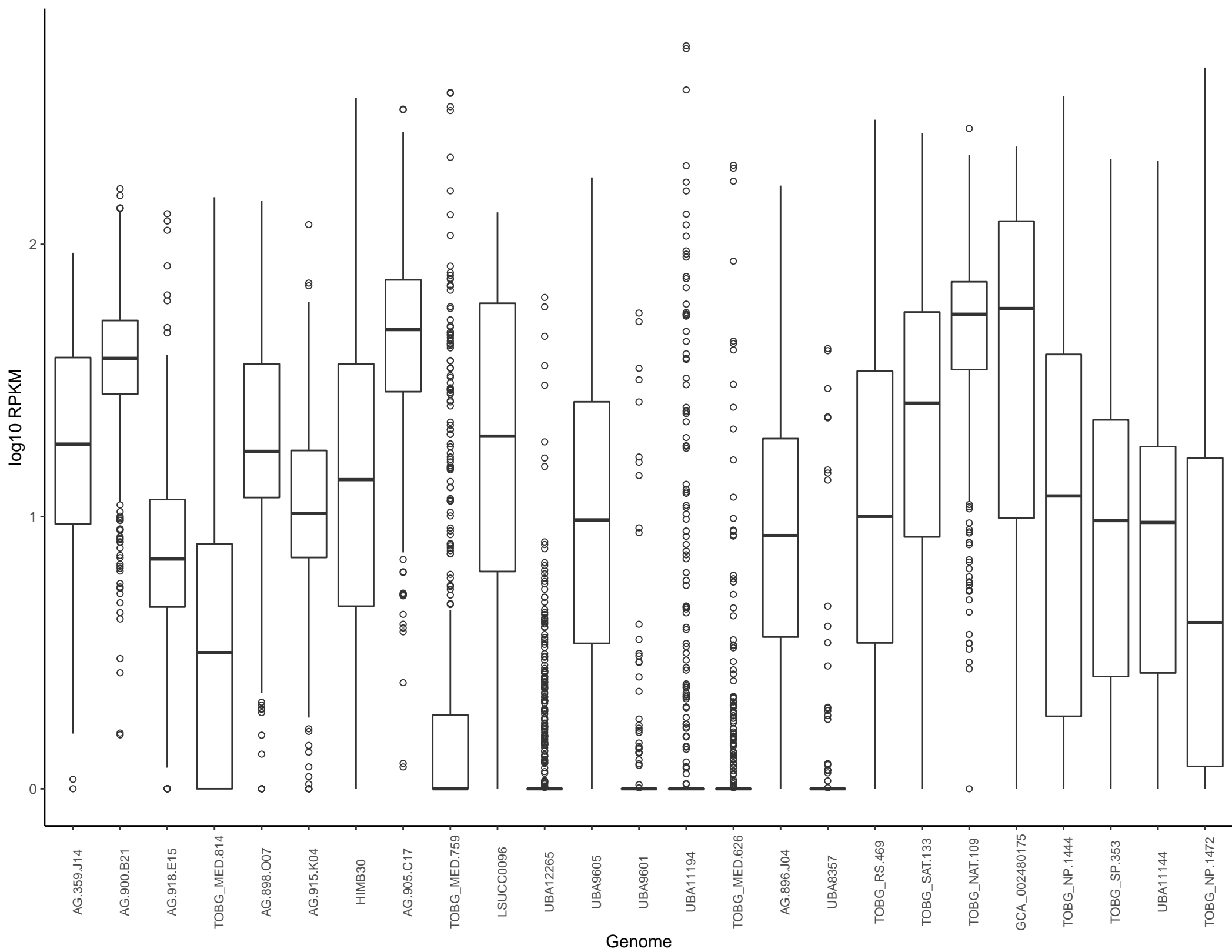

Supplement: FIG S4 [file msystems.00276-21-sf004.pdf]

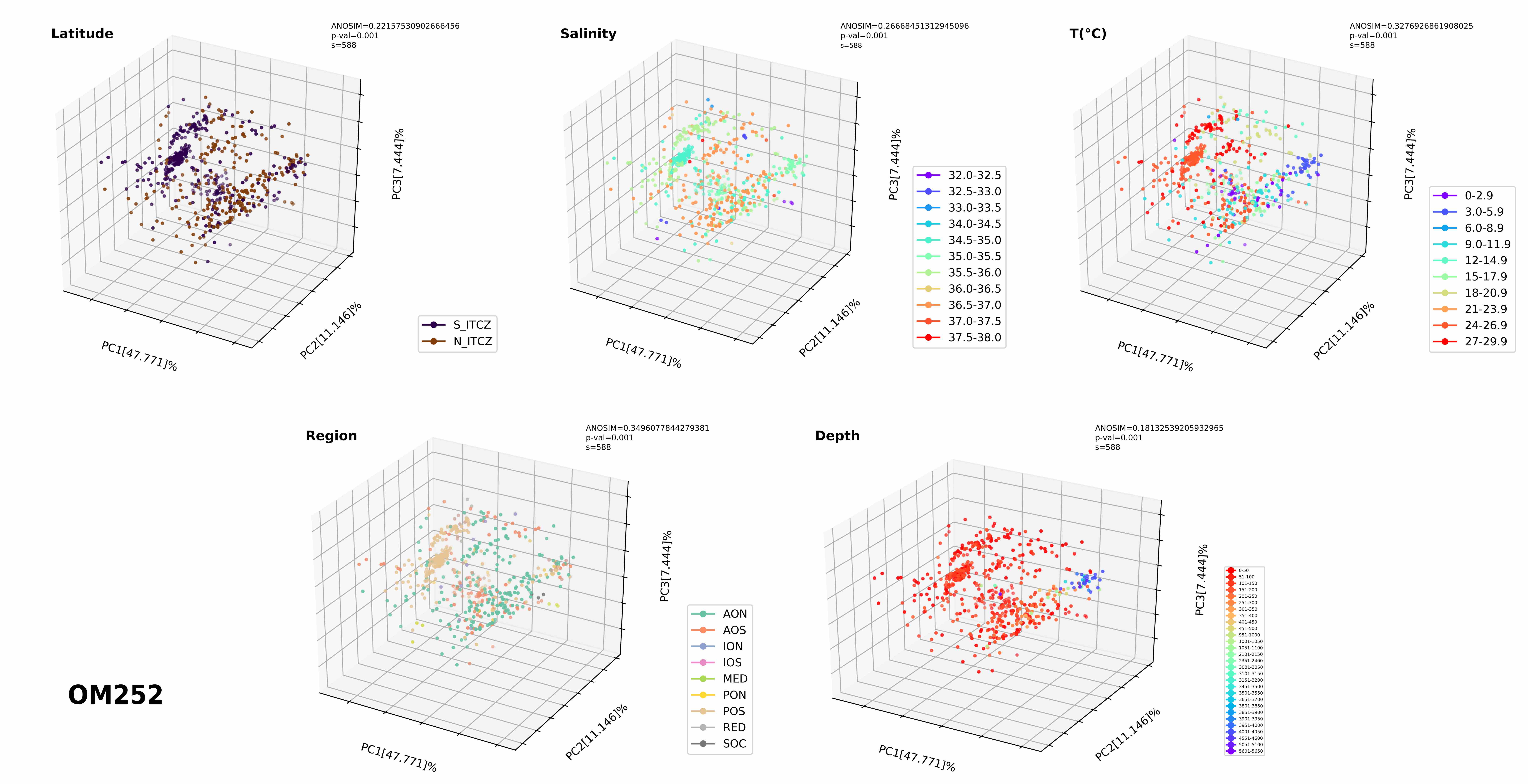

Supplement: FIG S5 [file msystems.00276-21-sf005.jpg]

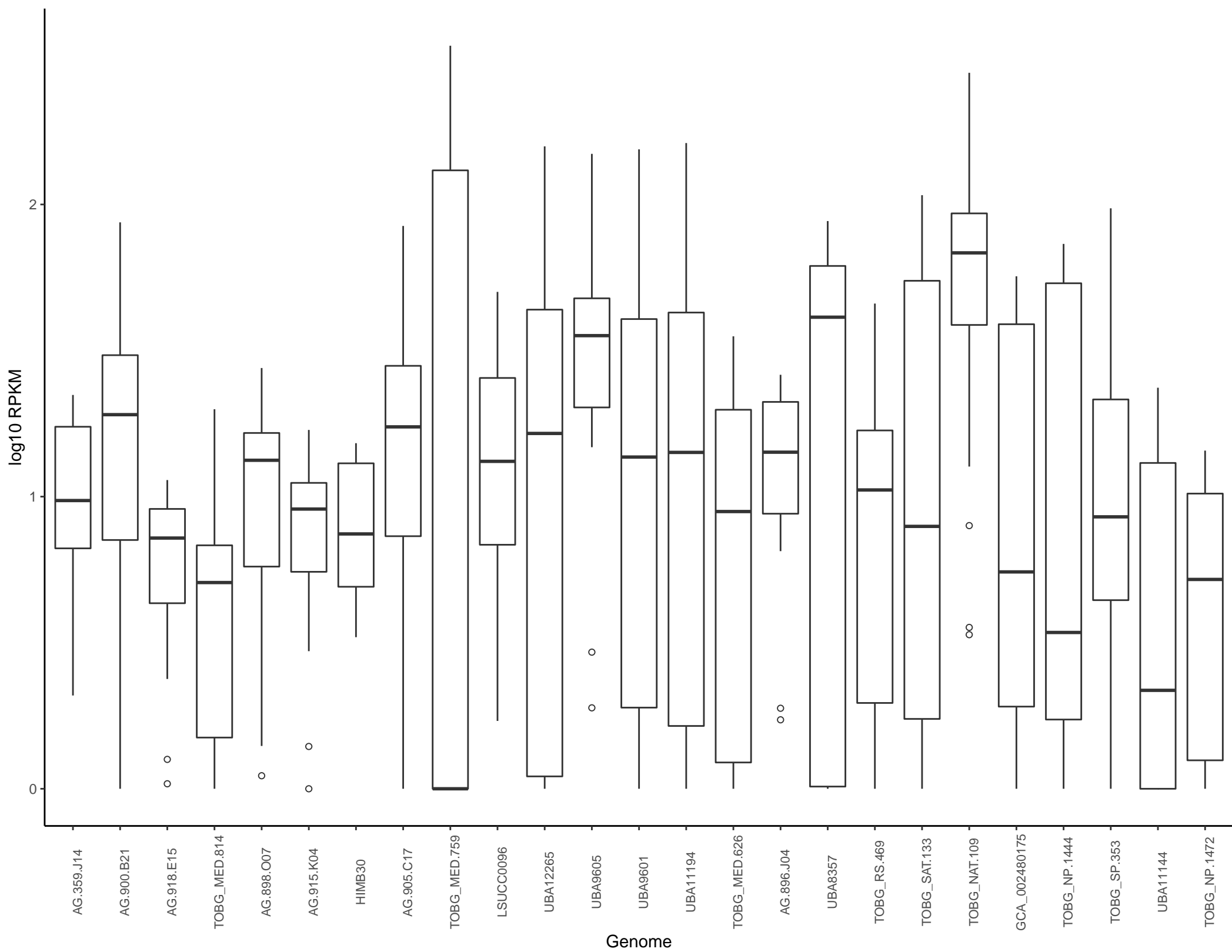

Supplement: FIG S6 [file msystems.00276-21-sf006.pdf]

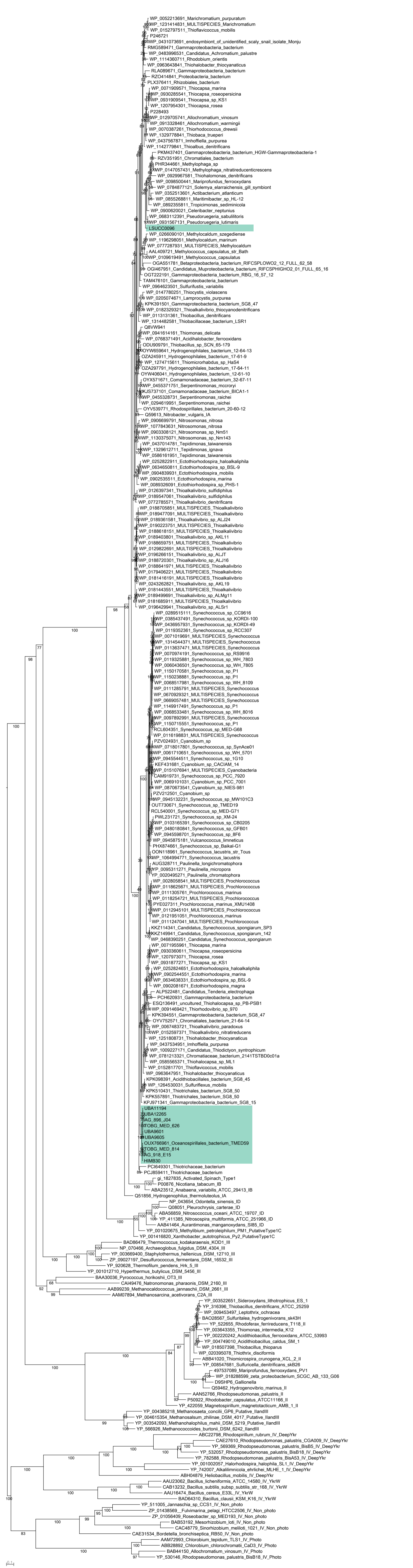

Supplement: FIG S8 [file msystems.00276-21-sf008.pdf]

A

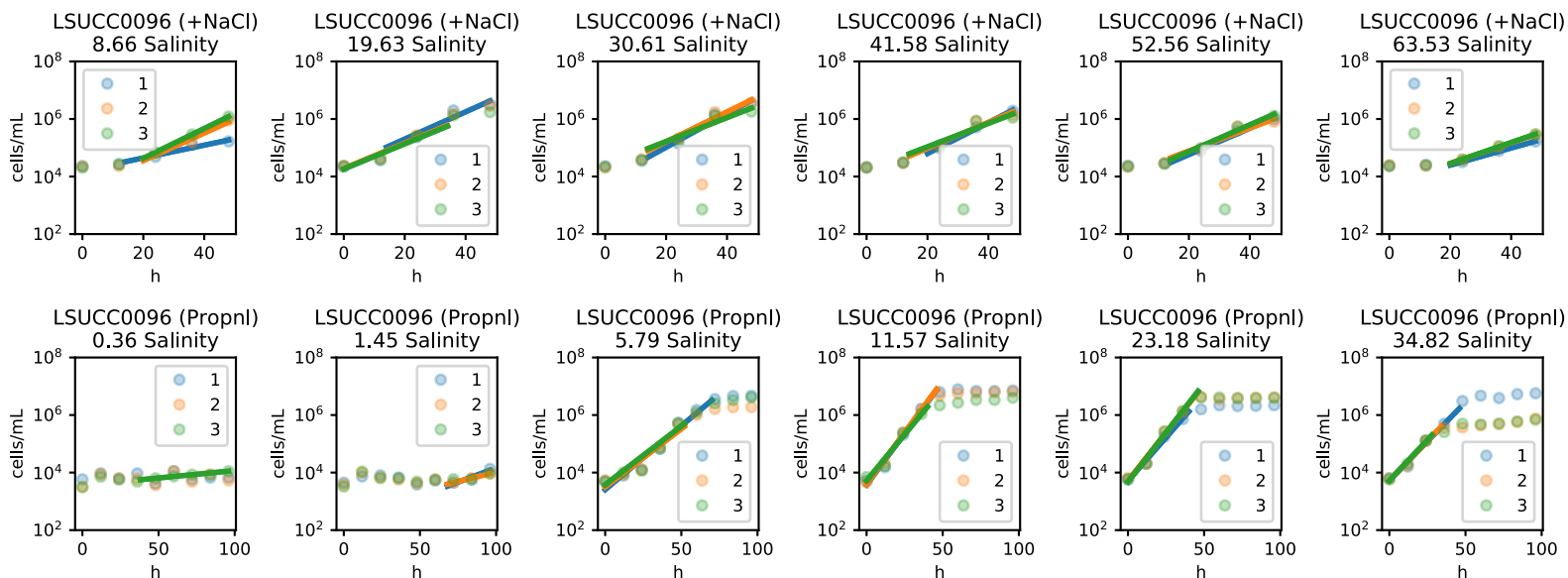

B

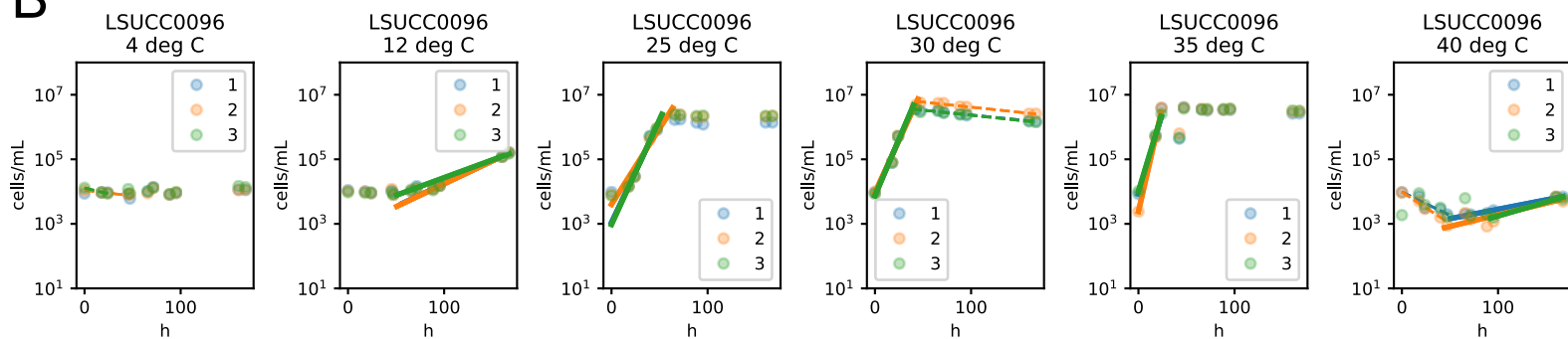

C

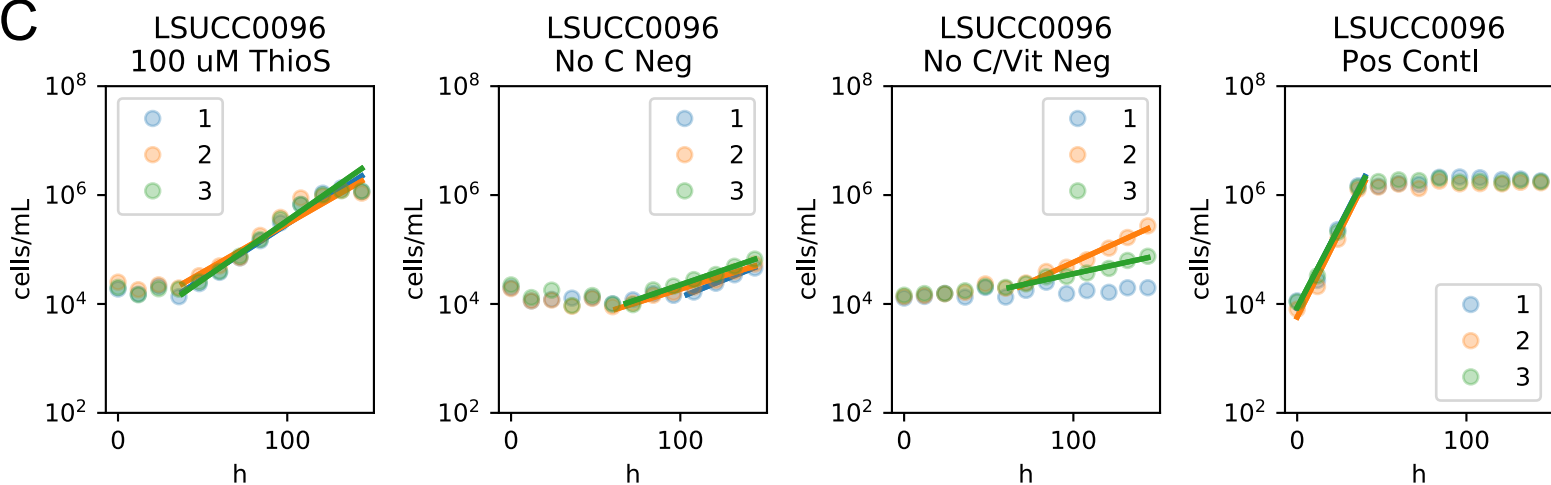

Supplement: FIG S9 [file msystems.00276-21-sf009.pdf]
