# Supplementary material for: Ecophysiology of the Cosmopolitan OM252 Bacterioplankton (Gammaproteobacteria)
Source: mSystems. 2021 Jun 29;6(3):e00276-21. doi: 10.1128/mSystems.00276-21 (PMC8269220; doi:10.1128/mSystems.00276-21)
Supplement: FIG S7 [file msystems.00276-21-sf007.pdf]

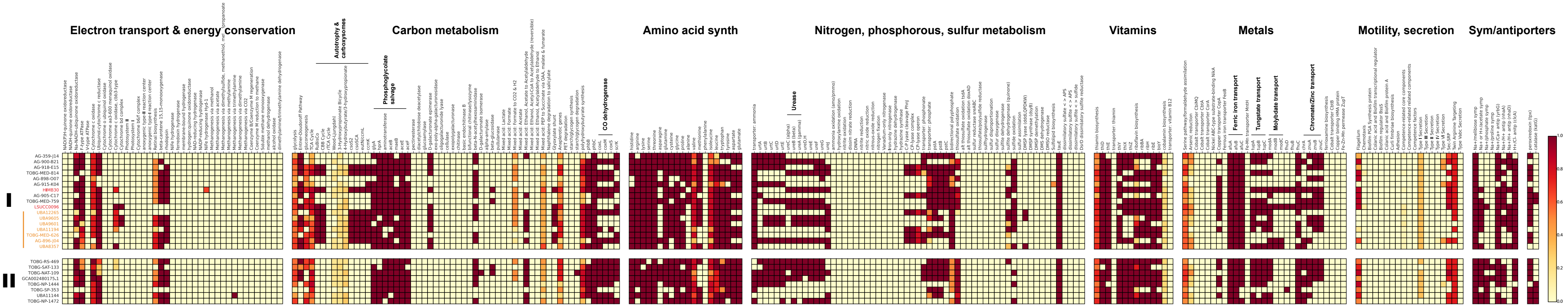
Electron transport & energy conservation
Carbon metabolism
Amino acid synth
Nitrogen, phosphorous, sulfur metabolism
Vitamins
Metals
Motility, secretion
Sym/antiporters
I
II
